# Comparative Analysis of ADR on China's National Essential Medicines List (2015 Edition) and WHO Model List of Essential Medicines (19th Edition)

**DOI:** 10.1155/2018/7862306

**Published:** 2018-06-10

**Authors:** Fangfang Zheng, Hongdou Chen, Yanfang Chen, Lu Ye, Huanhuan Wu

**Affiliations:** ^1^Suqian People's Hospital of Nanjing Drum-Tower Hospital Group, Suqian 223800, China; ^2^Affiliated Suqian Hospital of Xuzhou Medical University, Suqian 223800, China

## Abstract

**Objective:**

To explore the safety of the essential medicines recorded in China's list through the comparison of the list of essential medicines of China and the World Health Organization (WHO), as well as the analysis of the basic situation and characteristics of adverse drug reactions (ADRs) on the two essential medicines recorded in China's and WHO lists in order to provide a reference for the improvement of China's list.

**Methods:**

A retrospective descriptive study was conducted, based on the database in Jiangsu Province ADR Monitoring Center from 2013 to 2015. A total of 266869 cases reports were collected within this period, comparing the differences between the two essential medicines recorded in China's and WHO lists, considering number of ADRs, type of report, and modes of administration. Compare the differences between the two groups of drugs in the presence of new, severe, and new severe adverse events using chi square test.

**Results:**

Comparing the two essential medicines list, they have the same 117 species. When comparing ADRs in the two groups, most are antimicrobial, electrolytes, and acid-base balance drugs, regulate water, and are higher in China. In addition, with respect to the number of ADR types in the two groups, there is statistical significance (p<0.001) (total number is 68603 and 47515, new types are 12601 and 7262, the severe are 2714 and 7566, and the new severe are 820 and 716).

**Conclusion:**

Compared to the WHO list of essential drugs, China's list is still to be improved.

## 1. Introduction

Essential medicines satisfy people's desire for health; they are selected through cost-effectiveness comparison and according to the current situation, effectiveness, and safety of public health [1, 2]. The establishment of essential medicines list of China has been based on the directory of WHO [3, 4].

Although many reports which used the ADR database to advance the safety of drugs have been published [5, 6], there are also authors who based on the small sample of ADR of individual breeds analyzed essential medicines list of China [7]. These earlier findings lead us to other imperative, comprehensive, and updated questions. The goal of this study was to characterize the differences between the two essential medicines recorded in China's national essential medicines list (2015) [8] and WHO model list of essential medicines (19th edition) [9], considering classification, number of ADRs, type of report, and the way of administration during the period 2013-2015. The data comes from National Center for ADR Monitoring of Jiangsu Province.

## 2. Materials and Methods

China's national essential medicines list (2015 edition) and WHO model list of essential medicines (19th edition) were obtained from the ministry of health website. Using the database of Jiangsu Province ADR Monitoring Center from 2013 to 2015 to perform a retrospective study, we analyze the differences between the two essential medicines recorded in China's and WHO lists with ADR by the number, type of report, and the way of administration. Analyses employed descriptive statistics and chi square test.

## 3. Results

### 3.1. Comparison of the Occurrence of ADR with Medicines Recorded in China's and WHO Lists

There are 373 essential medicines recorded in WHO and 301 in China. The same species are 117. A total of 266869 cases of ADR were discharged within this period, with 76282 cases occurring in essential medicines recorded in China and 48310 cases in WHO. Among them, the most ADR are all anti-infective adjust water, electrolyte, and acid-base balance medicines. The second are respiratory medicines, medicines for pain and palliative care, and gastrointestinal medicines. The number of ADRs of these medicines in China's list is significantly higher than that in WHO (Table 1).

### 3.2. Comparison of the Type of ADR

It is presents the characteristics of the type of ADR discharged in the two essential medicines list (Table 2). Comparing the China's essential medicines list with number of ADRs with WHO, they have higher new and new severe adverse events (12601 and 820) and lower number of severe adverse events (2714). When comparing the type of new, new severe, and severe adverse events, all results are statistically significant (p<0.01).

### 3.3. Comparison of the Modes of Administration of ADR

**Figure 1:**
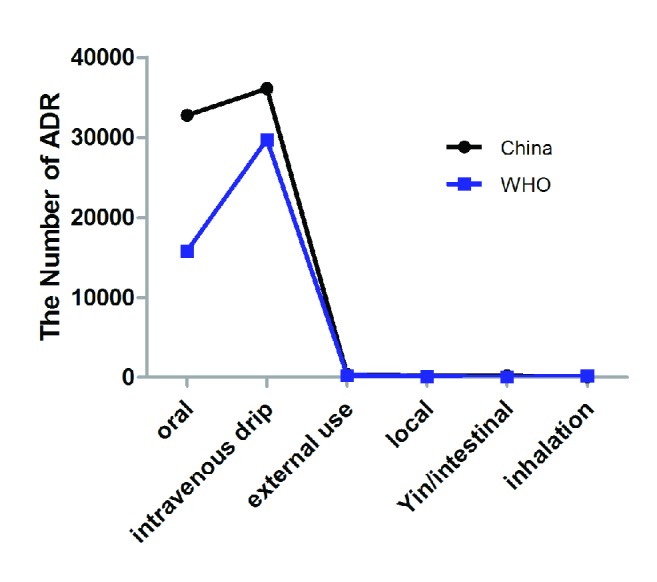
Administration route.

It is illustrate that oral, intravenous drip, external use, local, Yin/intestinal administration, and inhalation are the main modes of administration for adverse reactions to essential medicines recorded in China and WHO list (Figure 1). Also it was found that oral and intravenous drip modes of administration have the highest proportion, 47.0% and 51.8% in essential medicines list of China and 34.2% and 64.3% in WHO essential medicines list. The other modes of administration have a lower ratio.

## 4. Discussion

In recent years, the ADR reporting systems have been providing a basis for drug safety evaluation. There are authors who showed the necessity and feasibility of using big data to study the research on active monitoring of drugs [10, 11]. According to the China Food and Drug Administration (CFDA) “annual report on national drug adverse reaction monitoring” (2015), 1398, 000 ADR reports have been reported in nationwide. However, nearly 90,000 reports have been reported annually in Jiangsu Province, accounting for 6.4 percent of the national sample size. It can reflect the overall situation of the national ADR.

Therefore, exploring the safety of the drugs recorded in China's essential medicines list based on a large sample is necessary. The results showed that the number of ADRs in China's list was much higher than that of the WHO.

It is necessary to discuss the safety of the drugs recorded in China's essential medicines list based on the large sample data. The results showed that the number of ADRs in China's list was much higher than that of the WHO. Among them, anti-infective medicine and regulating water, electrolyte, and acid-base balance drugs have the most ADR (Table 1) and China was also higher than WHO. This is closely related to its usage amount, but we should pay more attention to irrational drug-induced ADR. The use of anti-infective drugs without indications, long course of treatment, and unreasonable preventive are very universal in clinical practice [12].

In addition, comparing from the type of report (Table 2), they have higher new and new severe and lower number of severe adverse events. Many factors can be associated with ADRs in it. Although 117 drugs are the same as recorded in the two lists, most are not the same. The ADRs caused by the different varieties of drugs in the two catalogues were identified as possible factors. In addition, many of the adverse reactions are associated with specific factors related to the patient and/or with the drug, like some drugs have a certain repeated type of ADR. Combined with China and WHO essential medicines list, drugs with more or severe ADR can be reevaluated and screened as a reference for screening of essential drugs in China [13].

In administration, our study shows that oral and intravenous drips have the highest proportion, in the two essential medicines lists. The other modes of administration have a lower ratio. The reason may be due to injection and injection of sterile powder compared with other medication, endotoxin, pH, osmotic pressure, and particles and other internal factors are more likely to lead to ADR in injection [14]. Oral administration is relative to a large number of applications [15].

Therefore, we should pay close attention to the patient's complaints and first-line clinical feedback of adverse reactions. For the revision of China's essential medicines list, an updated drug manual provides the most stringent basis [16].

## Figures and Tables

**Table 1:** The occurrence of ADR with medicines recorded in China's and WHO list.

Drug classification	WHO model list	China's list	The same number	WHO essential medicines		China essential medicines	
				ADR cases	Composition%	ADR cases	Composition%
Anti-infective	76	55	27	18128	37.52	24735	32.43
Anthelminthics	31	9	7	495	1.02	937	0.23
anaesthetics	13	4	2	455	0.94	28	0.03
Medicines for pain and palliative care	34	18	4	4089	7.65	6469	8.48
The nervous system	9	18	7	959	1.99	3116	4.08
Medicines used in mood disorders	14	6	3	2768	5.73	516	0.68
Cardiovascular	21	31	13	1958	4.05	6146	8.06
Respiratory medicines	5	8	1	229	0.47	3103	4.07
Gastrointestinal medicines	10	29	4	1673	3.46	4308	5.65
Urinary medicines	5	7	3	971	2.01	592	0.78
Medicines affecting the blood	22	16	4	657	1.36	1698	2.23
Endocrine medicines	23	21	4	1398	3.10	1941	2.54
Antiallergic medicines	5	5	1	983	2.04	1111	1.46
Immune system medicines	4	2	1	61	0.13	82	0.11
Vitamins, minerals	12	15	2	110	0.23	2156	2.83
Adjust water, electrolyte and acid base balance	9	9	6	11266	23.87	15762	20.66
Antidotes	15	6	3	203	0.48	132	0.17
Biological products	8	4	2	28	0.06	1098	1.44
Diagnostic agents	7	2	1	326	0.68	32	0.04
Dermatological medicines	16	12	9	730	1.51	418	0.55
Ophthalmic medicines	13	13	9	142	0.29	439	0.57
Ear and nose medicines	4	3	0	68	0.14	70	0.09
obstetrics and gynecology medicine	4	7	3	515	1.07	1260	1.65
Family planning medicines	13	1	1	98	0.20	133	0.17
	373	301	117	48310	100	76282	100

Note: repeat medication for one

**Table 2:** Comparetion the type of the ADR.

	Total		New		Sever		New severe	
	China	WHO	China	WHO	China	WHO	China	WHO
2013	23234	13705	4449	2253	932	2199	256	187
2014	21618	14882	4134	2280	877	2827	272	227
2015	23751	18928	4018	2729	905	2540	292	302
Totle	68603	47515	12601	7262	2714	7566	820	716

Note: using chi square test to compare the differences between the two groups of drugs in the presence of new, severe, and new serious adverse events; statistically significant (p<0.05). New: the adverse reactions are not specified in the drug specification. Severe: reaction to one of the following, damage caused by taking a drug: ① death; ② carcinogenic, teratogenic, or birth defects; ③ dangerous to life and can cause permanent or significant disability; ④ permanent damage to organ function; and ⑤ being hospitalized or staying in hospital for too long. New severe: the adverse reactions are severe and not specified in the drug specification.
